# Supplementary material for: Suboptimal Concentrations of Ceftazidime/Avibactam (CAZ-AVI) May Select for CAZ-AVI Resistance in Extensively Drug-Resistant Pseudomonas aeruginosa: In Vivo and In Vitro Evidence
Source: Antibiotics (Basel). 2022 Oct 22;11(11):1456. doi: 10.3390/antibiotics11111456 (PMC9686790; doi:10.3390/antibiotics11111456)
Supplement: Supplementary file 1 [file antibiotics-11-01456-s001.zip › antibiotics-1968782-supplementary.pdf]

Figure S1. Bland-Altman plot of observed and predicted ceftazidime concentrations for the four regimens in the overall experiments: Css of 12 mg/L, Css of 18 mg/L, Css of 30 mg/L and Css of 48mg/L. Css, steady-state concentrations; SD, standard deviations.

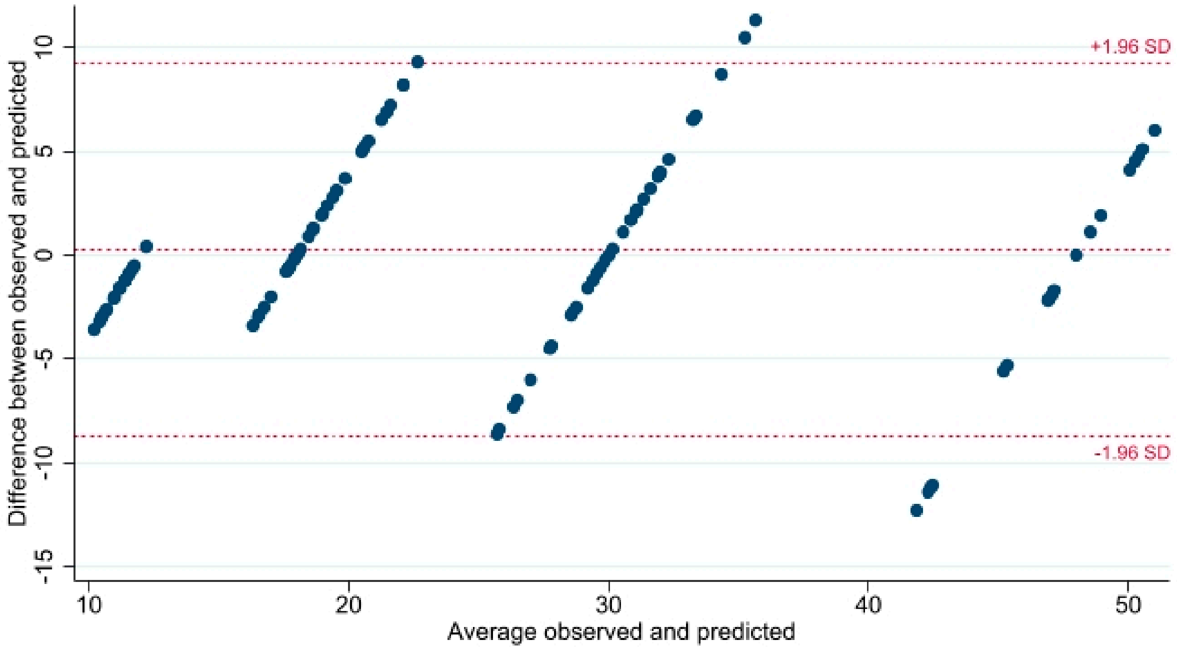

Table S1. Mean bacterial density (log10 CFU/mL) during 7-day HFIM assays and the emergence of CAZ-AVI resistant subpopulation onto agar supplemented with CAZ-AVI at 2X, 4X and 8X baseline MIC. CFU, colony forming unit; HFIM, hollow fiber infection model; CAZ/AVI, ceftazidime/avibactam; MIC, minimum inhibitory concentration; Css, steady-state concentrations. Data are presented as the mean concentration ± standard deviation.

	**0 h**	**8 h**	**24 h**	**48 h**	**72 h**	**96 h**	**144 h**	**168 h**
**Control**	**7.08±0.05**	**9.27±0.81**	**9.30±0.36**	**9.83±0.92**	**9.57±0.04**	**9.85±0.21**	**10.26±0.12**	**10.08±0.03**
2XMIC Drug Plates CAZ/AVI	0.00	0.00	0.00	0.00	0.00	0.00	0.00	0.00
4XMIC Drug Plates CAZ/AVI	0.00	0.00	0.00	0.00	0.00	0.00	0.00	0.00
8XMIC Drug Plates CAZ/AVI	0.00	0.00	0.00	0.00	0.00	0.00	0.00	0.00
**CAZ/AVI CI Css 12 mg/L (2XMIC)**	**7.08±0.05**	**9.11±0.31**	**7.65±0.88**	**9.70±0.09**	**9.60±0.24**	**7.48±0.15**	**7.81±0.06**	**8.65±0.52**
2XMIC Drug Plates CAZ/AVI	0.00	0.00	2.40±0.05	4.70±0.2	4.70±0.08	4.70±0.12	4.70±0.3	4.70±0.03
4XMIC Drug Plates CAZ/AVI	0.00	0.00	1.30±0.2	4.70±0.18	4.70±0.03	4.70±0.12	4.70±0.18	4.70±0.4
8XMIC Drug Plates CAZ/AVI	0.00	0.00	0.00	1.00±0.16	3.48±0.03	4.70±0.21	4.70±0.35	4.70±0.12
**CAZ/AVI CI Css 18 mg/L (3XMIC)**	**7.08±0.05**	**7.84±0.57**	**6.71±0.23**	**7.00±0.00**	**6.02±0.40**	**6.20±0.28**	**7.44±0.06**	**8.18±0.57**
2XMIC Drug Plates CAZ/AVI	0.00	0.00	0.00	1.70±0.24	4.70±0.15	4.70±0.03	4.70±0.4	4.70±0.14
4XMIC Drug Plates CAZ/AVI	0.00	0.00	0.00	0.00	3.48±0.2	4.70±0.31	4.70±0.26	4.70±0.10
8XMIC Drug Plates CAZ/AVI	0.00	0.00	0.00	0.00	0.00	4.70±0.05	4.70±0.12	4.70±0.28
**CAZ/AVI CI Css 30 mg/L (5XMIC)**	**7.08±0.05**	**6.93±0.21**	**5.25±0.42**	**5.33±0.21**	**3.78±0.17**	**3.88±0.41**	**3.48±0.37**	**2.92±0.11**
2XMIC Drug Plates CAZ/AVI	0.00	0.00	0.00	0.00	0.00	0.00	0.00	0.00
4XMIC Drug Plates CAZ/AVI	0.00	0.00	0.00	0.00	0.00	0.00	0.00	0.00
8XMIC Drug Plates CAZ/AVI	0.00	0.00	0.00	0.00	0.00	0.00	0.00	0.00
**CAZ/AVI CI Css 48 mg/L (8XMIC)**	**7.08±0.05**	**6.40±0.25**	**5.24±0.02**	**4.54±0.35**	**4.04±0.32**	**4.35±0.12**	**3.30±0.61**	**2.60±0.28**
2XMIC Drug Plates CAZ/AVI	0.00	0.00	0.00	0.00	0.00	0.00	0.00	0.00
4XMIC Drug Plates CAZ/AVI	0.00	0.00	0.00	0.00	0.00	0.00	0.00	0.00
8XMIC Drug Plates CAZ/AVI	0.00	0.00	0.00	0.00	0.00	0.00	0.00	0.00
